# In the shadows of snow leopards and the Himalayas: density and habitat selection of blue sheep in Manang, Nepal

**DOI:** 10.1002/ece3.6959

**Published:** 2020-11-23

**Authors:** Marc Filla, Rinzin Phunjok Lama, Tashi Rapte Ghale, Johannes Signer, Tim Filla, Raja Ram Aryal, Marco Heurich, Matthias Waltert, Niko Balkenhol, Igor Khorozyan

**Affiliations:** ^1^ Department of Conservation Biology University of Goettingen Goettingen Germany; ^2^ Third Pole Conservancy Kathmandu Nepal; ^3^ Wildlife Sciences Faculty of Forest Sciences and Forest Ecology University of Goettingen Goettingen Germany; ^4^ Institute of Medical Biometry and Bioinformatics Heinrich Heine University Duesseldorf Duesseldorf Germany; ^5^ Forest Research and Training Centre Babarmahal Kathmandu Nepal; ^6^ Chair of Wildlife Ecology and Management Faculty of Environment and Natural Resources University of Freiburg Freiburg Germany; ^7^ Department of Visitor Management and National Park Monitoring Bavarian Forest National Park Grafenau Germany

**Keywords:** Annapurna Conservation Area, bharal, *Panthera uncia*, predator‐prey, *Pseudois nayaur*

## Abstract

There is a growing agreement that conservation needs to be proactive and pay increased attention to common species and to the threats they face. The blue sheep (*Pseudois nayaur*) plays a key ecological role in sensitive high‐altitude ecosystems of Central Asia and is among the main prey species for the globally vulnerable snow leopard (*Panthera uncia*). As the blue sheep has been increasingly exposed to human pressures, it is vital to estimate its population dynamics, protect the key populations, identify important habitats, and secure a balance between conservation and local livelihoods. We conducted a study in Manang, Annapurna Conservation Area (Nepal), to survey blue sheep on 60 transects in spring (127.9 km) and 61 transects in autumn (134.7 km) of 2019, estimate their minimum densities from total counts, compare these densities with previous estimates, and assess blue sheep habitat selection by the application of generalized additive models (GAMs). Total counts yielded minimum density estimates of 6.0–7.7 and 6.9–7.8 individuals/km^2^ in spring and autumn, respectively, which are relatively high compared to other areas. Elevation and, to a lesser extent, land cover indicated by the normalized difference vegetation index (NDVI) strongly affected habitat selection by blue sheep, whereas the effects of anthropogenic variables were insignificant. Animals were found mainly in habitats associated with grasslands and shrublands at elevations between 4,200 and 4,700 m. We show that the blue sheep population size in Manang has been largely maintained over the past three decades, indicating the success of the integrated conservation and development efforts in this area. Considering a strong dependence of snow leopards on blue sheep, these findings give hope for the long‐term conservation of this big cat in Manang. We suggest that long‐term population monitoring and a better understanding of blue sheep–livestock interactions are crucial to maintain healthy populations of blue sheep and, as a consequence, of snow leopards.

## Graphical abstract

Blue sheep play a key ecological role in high‐altitude ecosystems and represent the staple prey for the globally threatened snow leopard. We show that the Manang area, Annapurna Conservation Area, Nepal, still maintains a relatively high density of these ungulates. Elevation and land cover are the main local determinants of blue sheep habitats.

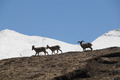

## 1 INTRODUCTION

Conservation initiatives have long been focused on rare and threatened species that face an imminent risk of extinction (Lindenmayer et al., 2011). However, common species are essential to secure functioning of species assemblages and ecosystems, and rare species may additionally rely on specific interactions with them. Therefore, even small declines in populations of common species may significantly disrupt natural processes (Gaston & Fuller, 2008). In addressing this issue, there is a growing agreement that conservation needs to be proactive and pay increased attention to common species and to the threats they face (Gaston & Fuller, 2008; Lindenmayer et al., 2011). Moreover, monitoring these populations is key for the early detection of population declines and for the evaluation and selection of conservation and management strategies (Frimpong, 2018; Gaston & Fuller, 2008; Waltert et al., 2008).

The blue sheep (*Pseudois nayaur*) is distributed from the Qilian Mountains in the north to the Himalayas in the south and is an example of a common species with a key ecological role in the mountain ecosystems of Central Asia (Harris, 2014). Due to its wide distribution, a presumably large global population, and a lack of documented severe population declines, the blue sheep is listed as a species of “Least Concern” on the IUCN Red List of Endangered Species (Harris, 2014). However, blue sheep and other mountain ungulates have been increasingly threatened by human pressures (Berger et al., 2013). Climate change, expansion of the human population, and infrastructure development are rapidly reducing blue sheep habitats (Aryal et al., 2016; Cui & Graf, 2009). Suitable habitats are not only being reduced but also degraded by co‐existing and competing livestock (Bhattacharya et al., 2020; Mishra et al., 2004; Suryawanshi et al., 2010) which also poses a risk for disease transmission (Dagleish et al., 2007; Gibb et al., 2020). In addition, illegal killing and legal hunting for subsistence or trophies (Aryal et al., 2015a; Aryal et al., 2010; Næss & Bårdsen, 2016) have a potential to harm local blue sheep populations.

The effects of these threats are alarming, especially considering the pivotal ecological role of blue sheep in low‐productivity high‐altitude ecosystems. Here, ungulates may affect plant species diversity and distribution through seed dispersal (Aryal et al., 2015b; Shrestha & Moe, 2015), and blue sheep represent the main wild prey for sympatric large carnivores, such as the globally vulnerable snow leopard (*Panthera uncia*; Lyngdoh et al., 2014). The snow leopard, which is a Himalayan flagship species, may heavily depend on blue sheep and selectively hunts these ungulates throughout their range (Aryal et al., 2014a; Lyngdoh et al., 2014; Shrestha et al., 2018). Therefore, protection of blue sheep populations is crucial to fulfill larger conservation goals such as maintaining ecosystem integrity and ecological functions, strengthening wildlife capacities to withstand increasingly difficult environmental conditions created by climate change, and fostering co‐existence between wildlife and local rural communities.

Although blue sheep persist in unprotected lands at reasonable densities, protected areas are likely to harbor population strongholds in several range countries (Harris, 2014). For instance, most suitable blue sheep habitats in Nepal are located within protected areas (Aryal et al., 2016) which accommodate relatively high densities of these ungulates (e.g., Shrestha & Wegge, 2008a). It is still a debate whether such abundance of wild prey would potentially reduce human–carnivore conflicts over livestock depredation (Chetri et al., 2017; Khorozyan et al., 2015) or increase them (Suryawanshi et al., 2017). Either way, detailed knowledge of habitat requirements by blue sheep is essential to identify and protect their key habitats and to support large and viable populations (Loehle & Li, 1996). Moreover, understanding blue sheep habitat preferences is also important to boost potential translocation programs. Recent calls for translocation programs to recover local blue sheep populations and mitigate human–snow leopard conflicts (Aryal et al., 2013, 2014b; Ferretti et al., 2014; Hanson et al., 2020) demand for the assessment of habitat quality in release sites (Wolf et al., 1998). Previous investigations of blue sheep were focused on species distribution (Aryal et al., 2016), habitat use and preferences (Aryal et al., 2014b; Bhardwaj et al., 2010; Khatiwada et al., 2007; Wegge, 1979; Wilson, 1981), resource partitioning and overlap with sympatric wild ungulates or livestock (Bhattacharya et al., 2020; Namgail et al., 2004, 2009; Shrestha & Wegge, 2008a; Suryawanshi et al., 2010), and foraging/bedding site selection (Liu et al., 2005a, 2007). Despite this, fine‐scale habitat requirements of blue sheep are still insufficiently studied. There is also limited knowledge about the main factors that shape blue sheep habitat selection. Based on this, it remains obscure whether blue sheep are more strongly affected by ecological or anthropogenic factors, which is relevant for conservation planning and management.

In this study, we aimed to estimate minimum blue sheep densities from total counts, determine population changes over time, and assess habitat selection for the Manang area of Annapurna Conservation Area, Nepal. Compared to other regions, this protected area harbored high blue sheep densities before and shortly after its official establishment (e.g., Sherpa & Oli, 1988 cited in Oli, 1991, 1994; Shrestha & Wegge, 2008a). The management strategy of the Annapurna Conservation Area follows an integrated conservation and development program aiming to achieve conservation goals and socio‐economic improvement, mainly through the implementation of ecotourism (Adams et al., 2004; Baral, 2013; Baral et al., 2019; Shrestha et al., 2010). This approach has been widely used throughout the blue sheep range (Nepal, 2002). As in other conservation programs, we consider blue sheep population trends in the study area as an important indicator of the effectiveness of such management strategies in regard to wildlife conservation (Ghoddousi et al., 2019; Waltert et al., 2008). We anticipate that a combination of our results with earlier studies (Bhattacharya et al., 2020; Oli, 1994; Shrestha & Wegge, 2008a, to name a few) will provide useful recommendations for the management and conservation of blue sheep also beyond the Annapurna region.

## 2 MATERIALS AND METHODS

### 2.1 Study area

**Figure 1:**
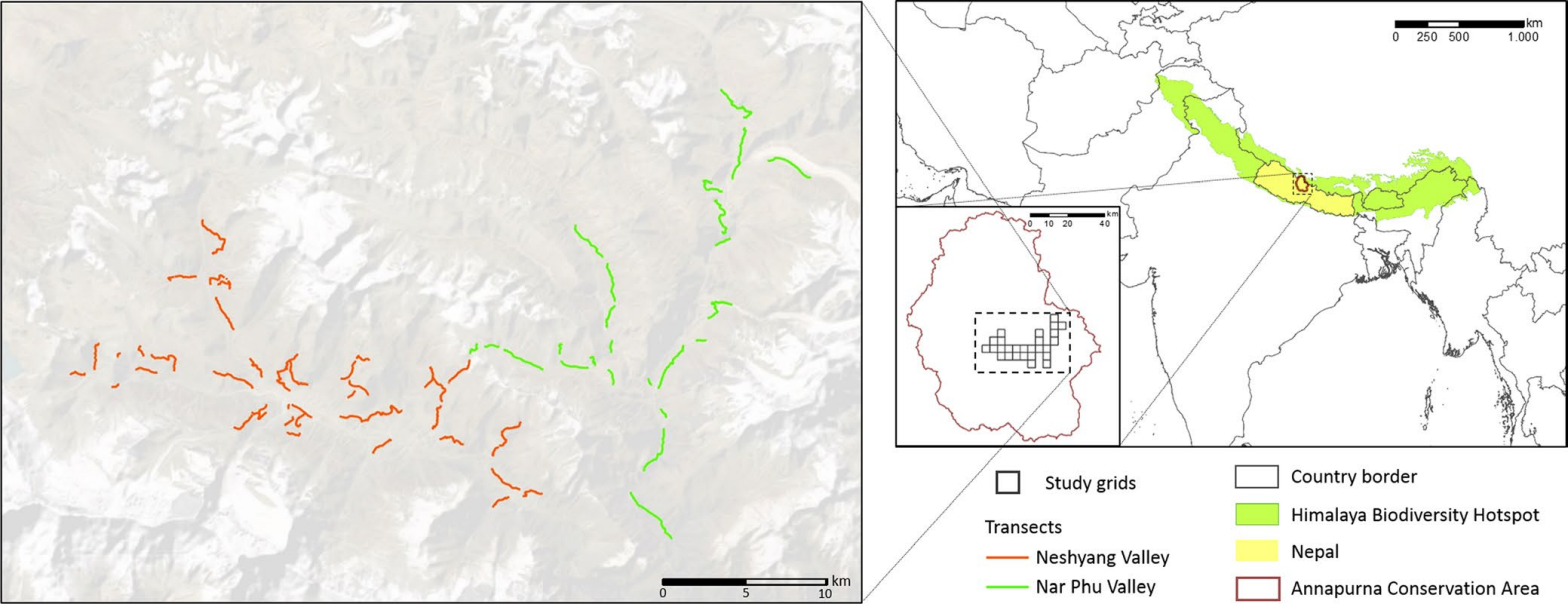
Location of Manang study area in the Annapurna Conservation Area within the Himalaya Biodiversity Hotspot (Created in ArcGIS^®^ 10.3.1). Sources: Esri, Digital Globe, GeoEye, Earthstar Geographics, CNES/Airbus DS, USDA, USGS, Aero Grid, IGN, and the GIS User Community; Conservation International, 2011 (Hotspot location); DIVA‐GIS, 2015 (country borders)

The Annapurna Conservation Area (ACA; IUCN management category VI) is located in the Himalaya Biodiversity Hotspot, covers an area of 7,629 km^2^, and forms the largest protected area in Nepal (Bhuju et al., 2007; Mittermeier et al., 2005; Figure 1). It provides habitats to at least 128 mammal, 514 bird, and more than 1,300 plant species, and hosts over 100,000 inhabitants (Baral et al., 2019). First tested in a single village development committee and in close cooperation with local people, the ACA was initiated in 1986 and officially gazetted in 1992 (Baral et al., 2019). This protected area has been managed through a long‐term participatory integrated conservation and development program by a nongovernmental organization, the National Trust for Nature Conservation. Local people are still allowed to live within the ACA boundaries, maintain traditional rights, and have access to natural resources. Financial resources generated from ecotourism and other sources have been invested in social capacity building, community development, and environmental education rather than in armed military guards (Baral et al., 2019).

The present study was carried out in the Manang area (28°35′3″‐28°50′11″N, 83°52′43″–84°20′16″E; Figure 1). Elevations of the effective study area (~450 km^2^) range from 2,870 m to 6,150 m above sea level (a.s.l.). Located in the rain shadow of the Annapurna Range, it is one of the driest areas in the Nepalese Himalaya, with the mean annual precipitation of ~400 mm and most precipitation falling as snow during winter (ICIMOD/MENRIS, 1995 cited in Aase & Vetaas, 2007; Chetri et al., 2017; Karki et al., 2015). Mean daily air temperatures range from less than −20°C in winter to slightly above 20°C in summer (Oli, 1991, Department of Hydrology and Meteorology, 1999 cited in Aase & Vetaas, 2007). Vegetation structure is determined by elevation and slope (Shrestha & Wegge, 2008a). Forests at lower elevations comprise the Himalayan white pine (*Pinus wallichiana*), East Himalayan fir (*Abies spectabilis*), Himalayan birch (*Betula utilis*), and black juniper (*Juniperus indica*; Ghimire & Lekhak, 2007). Above the timberline, vegetation is grouped into shrublands, alpine meadows, and alpine grasslands (Shrestha & Wegge, 2008a). The highest elevations are dominated by barren lands and permanent snowfields (Shrestha & Wegge, 2008a).

In spite of its harsh environmental conditions, the Manang area is relatively rich in mammalian species. Apart from the blue sheep, the Himalayan musk deer (*Moschus leucogaster*) and Himalayan tahr (*Hemitragus jemlahicus*) add to the ungulate community at lower elevations (Chetri et al., 2017). Several large and medium carnivores occur in the area including the snow leopard, Himalayan wolf (*Canis lupus chanco*), golden jackal (*Canis aureus*), and red fox (*Vulpes vulpes*; Chetri et al., 2017). The small mammal community is composed of such species as the Pallas's cat (*Otocolobus manul*), small mustelids (*Mustela* spp. and *Martes* spp.), and several species of voles (*Alticola* spp.) and pikas (*Ochotona* spp.; Oli, 1994, Chetri et al., 2017).

The livestock community includes sheep (*Ovis aries*), goats (*Capra hircus*), yaks (*Bos grunniens*), cattle (*B. taurus*), dzo (*B. grunniens* × *B. taurus*), mules (*Equus asinus* × *E. caballus*), and horses (*E. caballus*; Chetri et al., 2017). Livestock husbandry and crop farming are among the main subsistence economies in the area, with tourism being of increasing importance (Baral et al., 2019; Bhuju et al., 2007; Chetri et al., 2017).

### 2.2 Data collection

This study was based on two field surveys in late winter/spring (March–May 2019; hereafter referred to as spring) and in late summer/autumn (September–October 2019, hereafter referred to as autumn). To identify potential study units, we placed a grid of 4×4 km cells over the study area. This cell size compromised daily movements and home ranges of blue sheep (Schaller, 1967 cited in Garland, 1983; Wegge, 1976 cited in Jackson, 1996) and snow leopards (Jackson, 1996; Johansson et al., 2016). We further selected study units based on geographical and ecological living conditions of blue sheep (mean elevation 3,000–5,000 m and forest cover <50%; Aryal et al., 2014a; Harris, 2014) and also considered logistic challenges and accessibility (distance to settlements <10 km; Alexander et al., 2016). In selected grid cells, we placed transects along the features that are commonly used by blue sheep, snow leopards, and other wildlife and that typically provide good visibility over the surrounding area. These included riverbeds and ridgelines (Jackson, 1996; Suryawanshi et al., 2013), as well as other paths and potential connecting habitats. Such features were selected after consultation with local people and verification of terrain accessibility, and they were assumed to be unbiased to habitat preferences by blue sheep. Seasonal differences in transect lengths were predominantly due to terrain inaccessibility caused by unexpected snowfall.

We counted blue sheep mainly during the morning (6:00–10:00 a.m.) and afternoon hours (2:00–6:00 p.m.) when blue sheep activity is high (Liu et al., 2005b), by teams of 2–3 skilled observers including wildlife biologists and experienced local field assistants. We scanned adjacent ridgelines, slopes, gullies, and valleys by 10×32 binoculars (Kowa SV) and stopped regularly at suitable vantage points (Leki et al., 2018). Upon spotting blue sheep, we marked the observer position and measured the distance (m), angle (degree), and compass direction to the center of the detected animal cluster or individual (degree) using a handheld GPS (Garmin GPSMAP 64s), range finder (Leica Rangemaster CRF 1000‐R), and compass. Whenever possible, we classified blue sheep as adult males, adult females, and subadults (<2 years; Aryal et al., 2010). To avoid double counting, we noted unique features of individuals, such as broken horns and coloration patterns, and aimed at sampling adjacent grid cells on consecutive days (Leki et al., 2018). Blue sheep locations were plotted, verified, and modified in ArcGIS 10.3.1 (Esri, USA) and QGIS 3.4.8 (QGIS Development Team).

We used ten environmental predictor variables to analyze habitat use and selection by blue sheep: elevation, slope, terrain ruggedness, aspect, normalized difference vegetation index (NDVI), livestock presence, distance to cliff, distance to stream, distance to settlement, and distance to trail. Elevation was obtained from a digital elevation model of 30‐m resolution (DEM; ASTER Digital Elevation Model; NASA/METI/AIST/Japan Spacesystems and U.S./Japan ASTER Science Team, 2009). The DEM also served as a basis for the calculations of slope (degree; Horn, 1981), terrain ruggedness (m; Riley et al., 1999), and aspect (degree; Horn, 1981). For the analysis of habitat selection, we converted the aspect to the deviation of the surface orientation from the south. Thereby, we accounted for the ecological relevance of this variable (i.e., south‐facing slopes receiving most solar radiation) and prevented problems from fitting smoothing terms to a continuous variable with a circular orientation (i.e., 0°N is equal to 360°N).

In the absence of a fine‐scale land‐cover map, we used NDVI (Rouse et al., 1974), which quantifies vegetation greenness based on remote sensing data, as an indicator of land cover and a proxy for food availability. We applied the Annual Composite function in Google Earth Engine (Gorelick et al., 2017) to calculate the median annual NDVI from satellite images with adequate cloud cover. To link NDVI with actual land cover, we first classified land‐cover types at 203 predefined locations by satellite imagery and ground‐truthing. These included the grassland, shrubland, agricultural land, forest, settlement, barren land, water body, and permanent snowfield. We then extracted NDVI values at corresponding locations, assigned NDVI ranges to each land‐cover type, and applied the Wilcoxon rank‐sum test to check for differences in NDVI among land‐cover types (Figure S1, Table S1).

In addition, we assessed livestock presence (1)/absence (0) including large (yak, cattle, dzo, and horse) and small species (sheep and goat). We monitored and mapped livestock from transects using the same methodology as for blue sheep. We created 500‐m buffers around livestock locations to derive areas with livestock presence and absence during the survey. As blue sheep and livestock can graze together (R.P. Lama & M. Filla, personal observations), we considered this quite short buffer distance as reasonable to affect blue sheep–livestock interactions (see Table S2 for model outputs with different buffer widths). As various geographic features and human presence potentially influence blue sheep, we additionally calculated the distances to cliff (m), stream (m), settlement (m), and trail (m). Cliffs represent a potential escape cover, and we defined them as slopes exceeding 45° and larger than 90 m^2^ (Bhattacharya et al., 2020; Namgail et al., 2004). Streams were identified from DEM using the Fill, Flow Direction, and Flow Accumulation tools in ArcGIS. The actual stream network was adjusted and finalized through the comparison with rivers mapped in OpenStreetMap (https://download.geofabrik.de), ground‐truthing, and interpretation of satellite images. Locations of settlements were provided by governmental authorities (Survey Department, Government of Nepal, 2019), and we amended this layer by adding long‐term herder camps and by removing abandoned settlements and individual huts. Trails commonly used by tourists and/or local people were derived from OpenStreetMap (https://download.geofabrik.de). These trail locations were checked and modified by ground‐truthing and based on expert judgment.

### 2.3 Data analysis

In order to yield comparable estimates of blue sheep population size to previous studies in the study area, we extrapolated the minimum blue sheep density from total counts along the transects. Due to good visibility and often sparse vegetation at high elevations, we considered this approach as suitable to provide conservative estimates. We estimated this minimum density as the number of animals counted per area surveyed, irrespective of sex/age classes involved (Aryal et al., 2014b; Oli, 1994). Therefore, we summed up all blue sheep individuals observed within the buffers around transects. To obtain the actual survey area, we used the Visibility tool in ArcGIS and calculated the visible surface (viewshed) within the buffers of 1,000–1,500 m around the transects. The thresholds of 1,000 and 1,500 m were the maximum sighting distances considered in our surveys, that is, they outlined the areas that were scanned with the most reasonable effort. In fact, the 1,000‐m buffer included 93.5% and the 1,500‐m buffer included 99.0% of all blue sheep groups spotted along the transects. We manually corrected total surfaces by adding individual pixels (i.e., areas surveyed but categorized as “not visible”) and subtracting areas not sampled due to low visibility. We received the upper and lower limits of minimum density estimates by considering the adjusted 1,000‐ and 1,500‐m viewsheds, respectively.

We analyzed habitat use by blue sheep from all sightings in the study area, including incidental encounters. To analyze habitat selection, we compared actual blue sheep presence sites along the transects with available sites. In order to sample available sites, we first created a large number of random pseudo‐absence points (*n* = 50,000) in the 1,500‐m viewshed around the transects. We then sampled from these points with the probabilities obtained from a density function of an exponential distribution parameterized with the observed distances of blue sheep to the transects (rate_spring_ = 0.0035; rate_autumn_ = 0.0024). As recommended by Barbet‐Massin et al. (2012), we drew 100 times as many points as we had field observations for each survey to gain a good model performance based on approximately 10,000 pseudo‐absence points per season.

We randomly attributed observed group sizes to pseudo‐absence points in spring as we did not find a significant correlation between the group size and the distance to the transect in this season (Pearson's *r* = −0.090, *p* = .389). In contrast, these parameters were positively correlated in autumn (Pearson's *r* = 0.242, *p* = .012). Therefore, we fitted a linear model where we explained the group size as a function of the intercept and the distance to the transect. The coefficients were 8.498 ± 1.893 (*p* < .001) for the intercept and 0.009 ± 0.003 (*p* = .012) for the distance to the transect.

We investigated the effects of the above‐mentioned environmental predictor variables on habitat selection by blue sheep using generalized additive models (GAMs). GAMs have been increasingly used in habitat selection analyses (e.g., Dupke et al., 2017; Liang et al., 2017; Rayment et al., 2015, to name a few), they are rather flexible and capable of modeling nonlinear relationships, which is appropriate for ecological datasets (Guisan et al., 2002), and we expected them to optimally fit various predictor variables. Blue sheep presence (1)/pseudo‐absence (0) served as the binary response variable in models separated for spring and autumn. Due to seasonal fluctuations and regular fission–fusion changes in group composition and size (Schaller, 1973 cited in Harris, 2014; Oli, 1996; Wang & Hoffmann, 1987), we treated each observation of single animals or groups as independent and adjusted for the number of adults by weighting. In the weighting process, we did not change the total number of observations in order not to erroneously increase the sample size. Each observation was assigned the weight as the number of adults divided by the total number of adults and multiplied by the total number of observations. The number of adult blue sheep was defined as the number of identified adults added by the number of unidentified individuals multiplied by the ratio of adults among all classified individuals. As recommended by Barbet‐Massin et al. (2012), we attributed the same total weight to pseudo‐absence points as to presence locations. We examined multicollinearity between predictor variables before modeling. Either of two variables was excluded if the absolute value of Pearson's correlation coefficient was equal to or greater than 0.7 (Dormann et al., 2013). Thus, we excluded terrain ruggedness which was highly correlated with slope (*r*_spring_ = 0.948, *r*_autumn_ = 0.945). We decided to retain the slope due to its better comparability across the studies (e.g., Aryal et al., 2014b). We further excluded livestock presence from spring models due to its high correlation with the distance to settlement (*r*_spring_ = −0.721). We retained the distance to settlement since most livestock is gathered around settlements in late winter/early spring and the locations of settlements were complete, yet some livestock could go undetected. Blue sheep observations in forested areas were omitted from GAMs as we assumed a significantly lower detection probability in this land‐cover type (see Table S3, Figure S2 for model outputs with forested areas included).

We analyzed the relative importance of variables through a random permutation procedure. We randomized one variable and then calculated the correlation between the predictions made by the randomized and original models (Thuiller et al., 2009). For each variable, we repeated this procedure 100 times to account for random effects. Then, we calculated a raw importance value for each variable as one minus mean correlation between the predictions made by the original and randomized models (Thuiller et al., 2009). Eventually, we standardized the relative importance values to the sum of one.

We performed sensitivity analyses by repeatedly modifying various assumptions and parameters, such as the location of random pseudo‐absence points and inclusion/exclusion of forested areas. Modification of these parameters did not change the main model outputs (Table S3, Figures [Link], [Link], [Link], [Link]). We conducted data processing and statistical analyses in R (R version 3.6.0; R Core Team, 2019), unless otherwise indicated. The following R packages were used: Distance (Miller et al., 2019), dplyr (Wickham et al., 2019), ggplot2 (Wickham, 2016), gratia (Simpson, 2020), MASS (Venables & Ripley, 2002), mgcv (Wood, 2011), polycor (Fox, 2019), raster (Hijmans, 2019), readxl (Wickham & Bryan, 2019), rgdal (Bivand et al., 2019), rgeos (Bivand & Rundel, 2019), sf (Pebesma, 2018), and sp (Pebesma & Bivand, 2005). We used standard error (SE) as a measure of variation.

## 3 RESULTS

### 3.1 Population density

We covered 60 transects of a total length of 127.9 km in spring (mean: 2.1 ± 0.2 km/transect) and 61 transects of a total length of 134.7 km in autumn (mean: 2.2 ± 0.2 km/transect). Altogether, we spotted 1,905 blue sheep (143 observations) during the fieldwork in spring and 2,058 blue sheep (146 observations) during the fieldwork in autumn. Thereof, 1,408 individuals (94 observations) were spotted along the transects in spring and 1,648 individuals (108 observations) in autumn. Blue sheep group size ranged from single animals to 86 individuals in spring and to 113 individuals in autumn. Mean group size was 14.4 ± 1.3 individuals in spring and 14.4 ± 1.5 individuals in autumn.

A total of 1,387/1,408 and 1,419/1,606 blue sheep were spotted within 1,000/1,500 m from the transects in spring and autumn, respectively. The conservative extrapolation of minimum blue sheep densities yielded 6.0–7.7 individuals/km^2^ and 6.9–7.8 individuals/km^2^ over the surveyed area (180–234 km^2^) in spring and autumn, respectively (see Table S4 for density estimates based on alternative maximum sighting distances). These estimates were higher in Nar Phu Valley (7.6–10.0 individuals/km^2^ in spring and 7.9–9.4 individuals/km^2^ in autumn) than in Neshyang Valley (4.6–5.9 individuals/km^2^ in spring and 6.0–6.6 individuals/km^2^ in autumn; Figure 1; see Table S5 for fine‐scale density estimates).

### 3.2 Habitat use and selection

**Figure 2:**
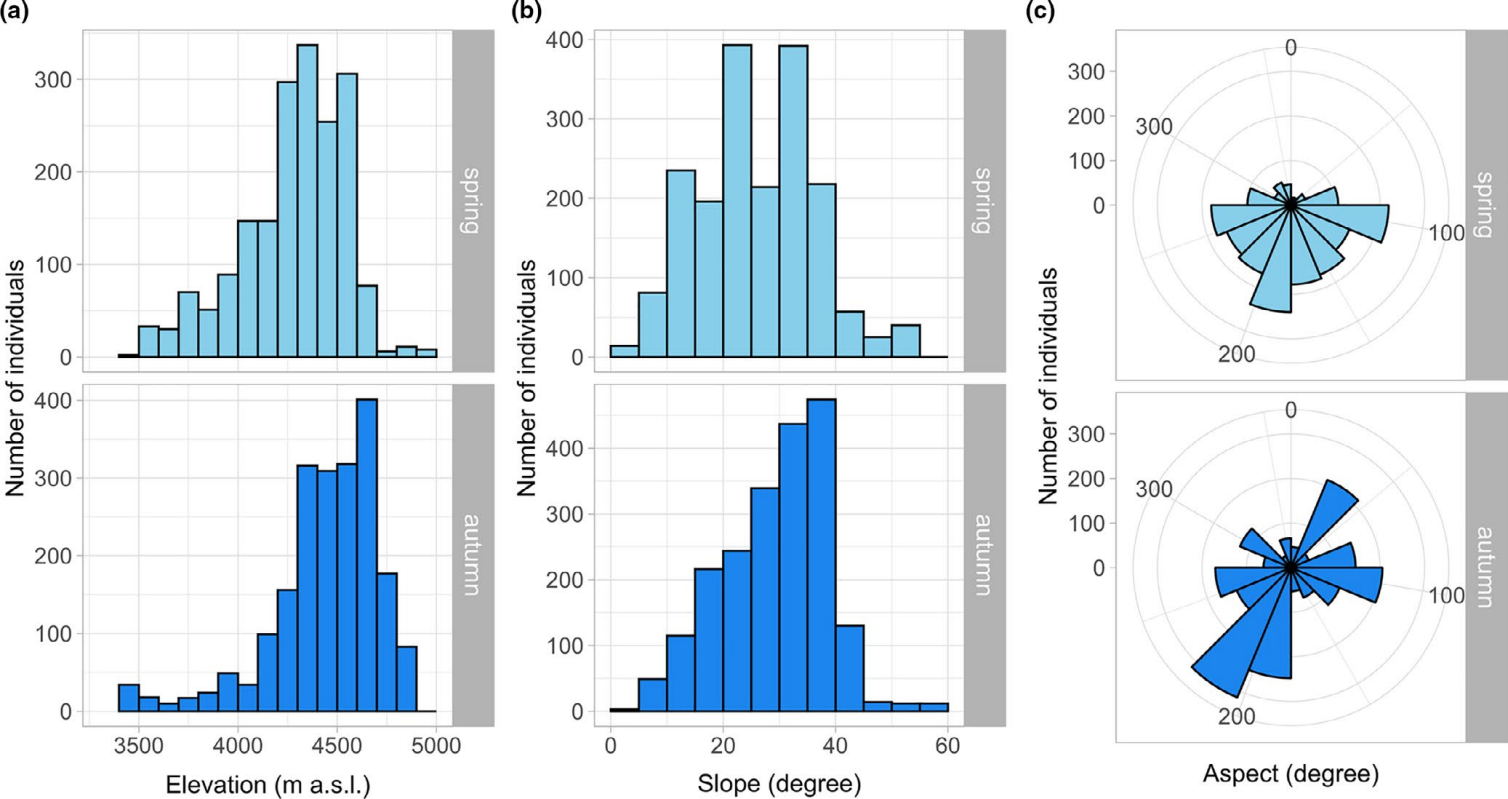
Habitat use by blue sheep in Manang. Shown are the frequencies of elevations (a), slopes (b), and aspects (c) used by blue sheep based on direct observations in spring and autumn

Blue sheep were sighted between 3,440 m and 4,958 m a.s.l., though pellets indicated their presence also at higher elevations around 5,100 m (R.P. Lama & M. Filla, unpublished data). The majority of individuals was observed at 4,200–4,600 m a.s.l. in spring (mean: 4,276 ± 6 m a.s.l.) and 4,300–4,700 m a.s.l. in autumn (mean: 4,443 ± 6 m a.s.l.; Figure 2). This seasonal difference in altitudinal use by blue sheep was significant (Wilcoxon rank‐sum test: W = 1,150,206, *p* < .001). In both seasons, blue sheep used a gradient of slope declivity ranging from flat terrain to steep cliffs (>50°), but most animals were spotted in moderately rugged terrain to rather strong slopes (Figure 2). Blue sheep used mainly southern slopes in spring, whereas this pattern was less distinct in autumn (Figure 2).

We spotted blue sheep in various land‐cover types including grasslands, shrublands, agricultural lands, barren lands, snowfields, and open forests. In spring, the majority (77.5%) of animals used habitats having NDVI values between 0.25 and 0.5 and associated mainly with grasslands, shrublands, and agricultural lands (see Figure S1). The same applied to autumn (55.0%), though the use of less vegetated habitats increased in this season. Overall, the NDVI values of habitats used by blue sheep did not differ between spring and autumn (Wilcoxon rank‐sum test: W = 1,921,320, *p* = .684).

Blue sheep observed from the transects were rarely encountered close to villages: 2.8% of adults were <200 m away from settlements and 7.9% were <500 m away, in comparison with 5.2% and 16.9% of randomly chosen adults. In contrast, we regularly spotted blue sheep close to hiking trails and streams: 25.7% and 18.1% of adult blue sheep observed from the transects were <200 m away from hiking trails and streams, respectively (Figure S6). In addition, 27.8% of adult blue sheep observed from the transects were spotted close (<500 m) to livestock in spring, while this applied to only 17.3% of individuals in autumn.

The GAMs fitted to model habitat selection by blue sheep were capable of explaining 19.0% and 27.0% of the deviance in spring (*n* = 8,927, adjusted *R* ^2^ = 0.159) and autumn (*n* = 10,283, adjusted *R* ^2^ = 0.200), respectively. Elevation and land cover were the only significant variables (*p* < .05) for habitat selection by blue sheep in both seasons (Table 1).

**Table 1:** Summary of generalized additive models (GAMs) describing habitat selection by blue sheep in Manang based on direct observations in spring and autumn. The estimates of the coefficient, standard error (SE), *z*‐values (z), and *p*‐values (p) are shown for categorical variables (not given for spring due to multicollinearity). The estimated degrees of freedom (edf), residual degrees of freedom (Ref.df), chi‐square test statistics (χ^2^), and *p*‐values (p) are given for continuous variables

Variables	Spring				Autumn			
Categorical variables								
	Coefficient	**SE**	**z**	**p**	Coefficient	**SE**	**z**	**p**
(Intercept)	−0.662	0.222	−2.985	.003	−1.003	0.303	−3.316	.001
Livestock	–	–	–	–	−0.444	0.436	−1.019	.308

**Continuous variables**								
	**edf**	**Ref.df**	**χ^2^**	**p**	**edf**	**Ref.df**	**χ^2^**	**p**
Elevation	2.250	2.888	12.832	.006	2.740	3.499	21.939	<.001
Slope	1.000	1.001	1.141	.286	3.580	4.491	6.323	.199
Aspect	1.017	1.034	2.428	.127	1.000	1.001	0.011	.918
NDVI	2.181	2.775	11.475	.008	1.000	1.000	10.773	.001
Cliff	1.000	1.000	0.452	.502	1.000	1.000	0.049	.825
Stream	1.087	1.169	0.012	.903	1.927	2.457	2.071	.403
Settlement	1.000	1.000	0.611	.435	1.000	1.000	0.044	.835
Trail	1.001	1.002	0.000	.995	1.001	1.001	0.182	.670

**Figure 3:**
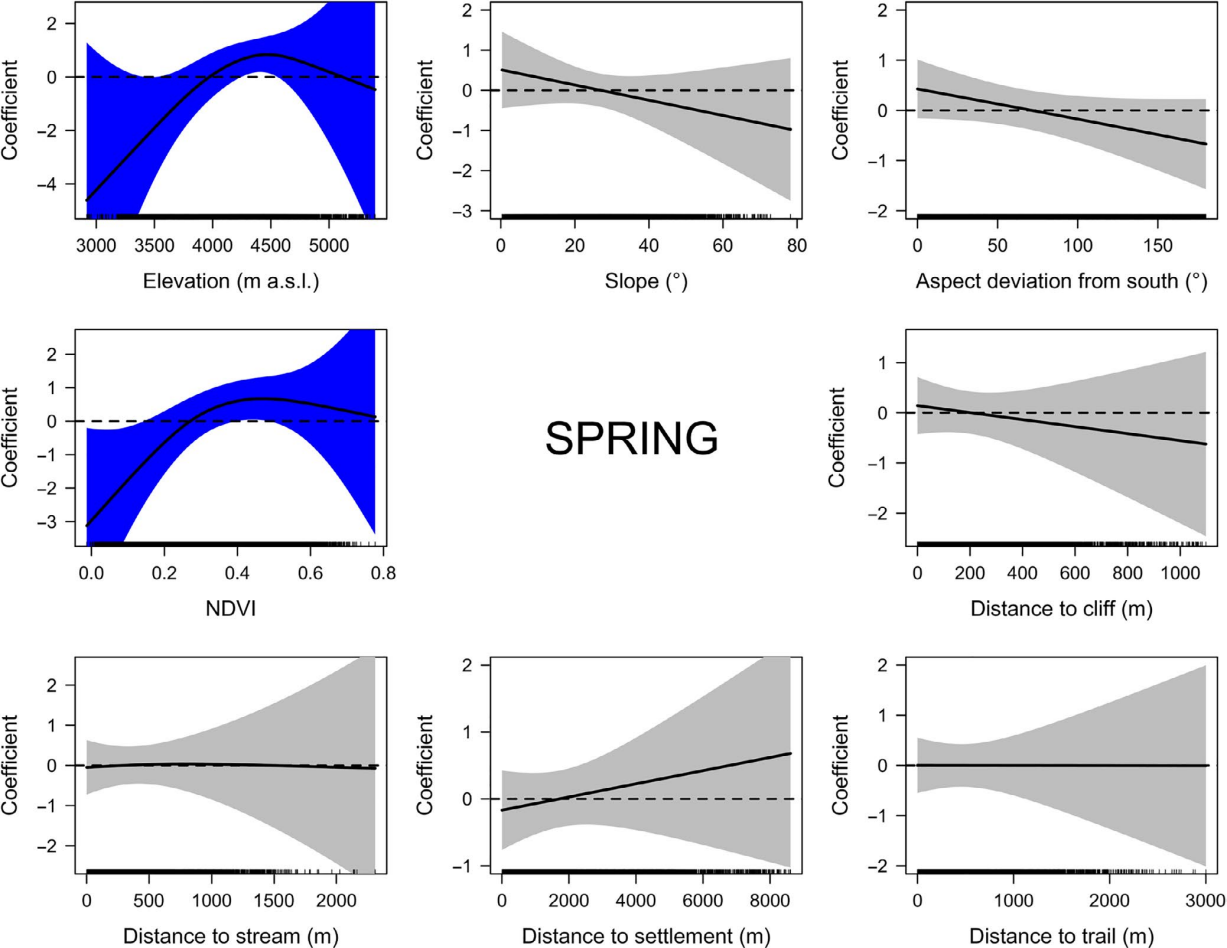
Plots of generalized additive models (GAMs) describing habitat selection by blue sheep in Manang based on direct observations in spring. The confidence intervals of significant variables are blue

In spring, blue sheep preferred elevations between 4,250 m and 4,550 m a.s.l. (Figure 3). Moreover, they selected land‐cover types having NDVI values associated mainly with grasslands, shrublands, and agricultural lands (NDVI = 0.40–0.49) and avoided land‐cover types with NDVI values associated with less vegetated habitats (i.e., barren lands, glaciers, and water bodies; NDVI < 0.14; Figure 3 and Figure S1, Table S1).

**Figure 4:**
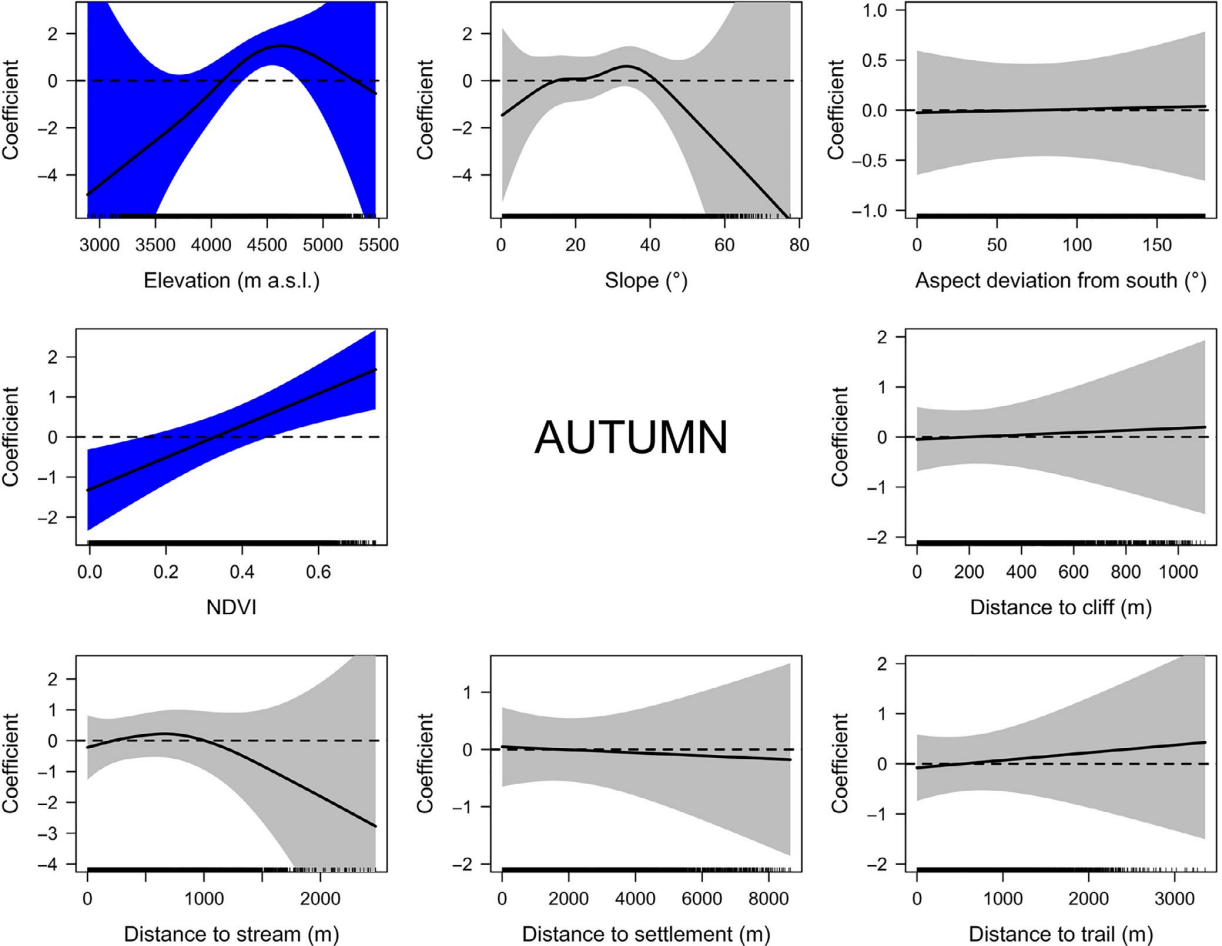
Plots of generalized additive models (GAMs) describing habitat selection by blue sheep in Manang based on direct observations in autumn. The confidence intervals of significant variables are blue

In autumn, blue sheep selected elevations between 4,300 m and 4,800 m a.s.l. (Figure 4). Besides, animals avoided areas with little vegetation (barren lands, permanent snowfields, and water bodies; NDVI < 0.14) and preferred land‐cover types having NDVI values associated mainly with grasslands and agricultural lands (NDVI > 0.47; Figure 4 and Figure S1, Table S1).

**Figure 5:**
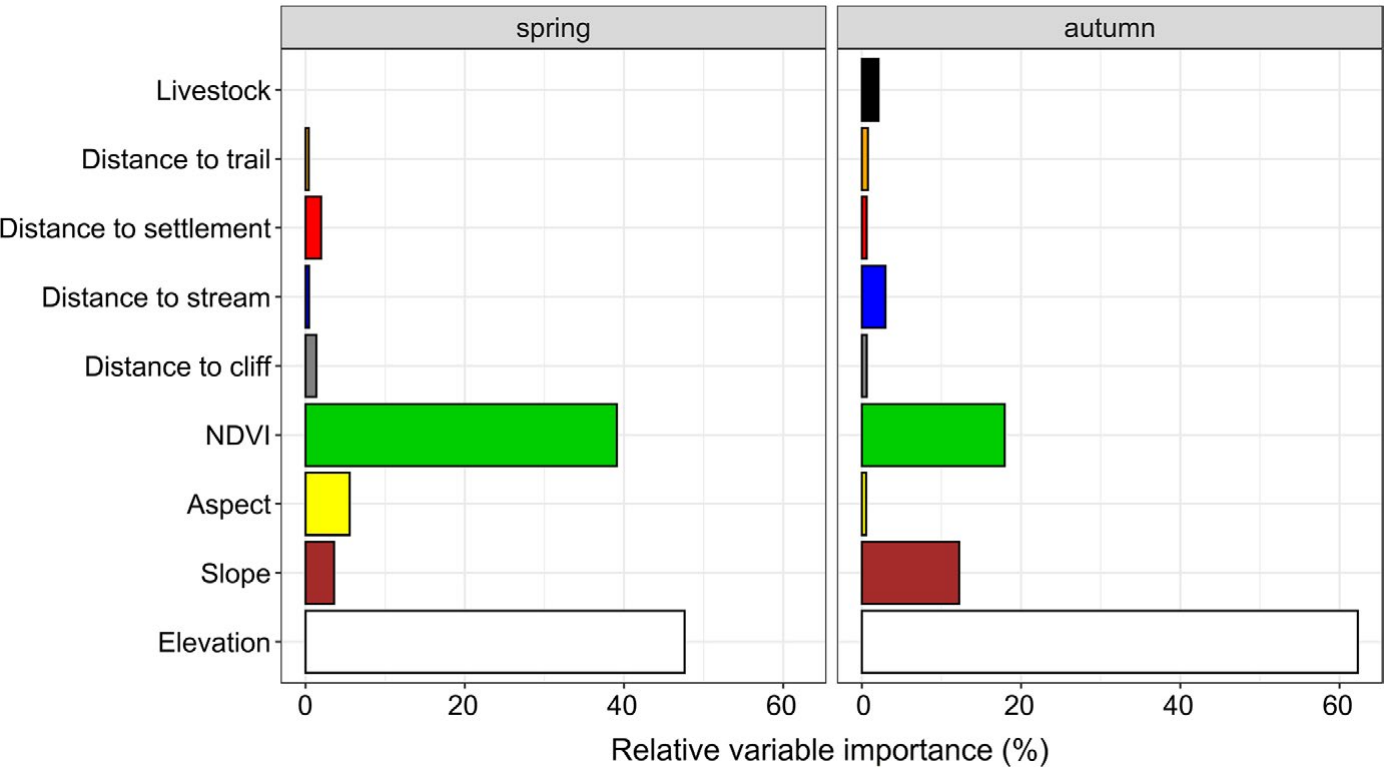
Relative variable importance (%) in generalized additive models (GAMs) describing habitat selection by blue sheep in Manang based on direct observations in spring and autumn

The importance of variables varied among seasons. Elevation and, to a lesser extent, land cover shaped blue sheep habitat selection (Figure 5). Elevation had the strongest explanatory power in both seasons (spring: 47.6%; autumn: 62.3%), whereas land cover was important in spring (39.1%) but much less so in autumn (17.9%).

## 4 DISCUSSION

This study shows that now, almost thirty years after the official establishment of the Annapurna Conservation Area, blue sheep densities in Manang are still rather high. Habitat selection by this ungulate is primarily driven by elevation and, to a lesser extent, by land cover indicated by NDVI, whereas the considered anthropogenic variables apparently have limited effects. Blue sheep tend to occur at higher elevations in autumn than in spring and select habitats associated with green vegetation including grasslands and shrublands.

The main purpose of this study was to estimate the minimum blue sheep density in Manang which would be comparable with minimum densities of this species in other areas and time periods. We applied total counts which, apart from producing such minimum density estimates, are also cost‐effective and reliable for the analysis of population trends in mountain ungulates (Largo et al., 2008; Loison et al., 2006). Our result (6.0–7.7 individuals/km^2^ in spring and 6.9–7.8 individuals/km^2^ in autumn) is similar to previously reported densities in the Manang area both before and after the official establishment of the Annapurna Conservation Area (before: 6.6–10.2 individuals/km^2^; Sherpa & Oli, 1988 cited in Oli, 1991; Wegge & Oli, 1988 cited in Oli, 1991; Oli, 1994; after: 6.3–9.4 individuals/km^2^; Thapa, 2005; Shrestha & Wegge, 2008a; Wegge et al., 2012) and might compromise the alarming result of Aryal et al. (2014b; 2.1 individuals/km^2^).

When compared with similar studies in other parts of the species range, the minimum blue sheep densities in Manang can be considered as being relatively high. For instance, in other parts of the Nepalese Himalaya the minimum densities were estimated as 0.5–4.2 individuals/km^2^ in Upper Mustang in the Annapurna Conservation Area (Aryal et al., 2014b; WWF Nepal, 2013; R.P. Lama & M. Filla, unpublished data), Dhorpatan Hunting Reserve (Aryal et al., 2010; Wilson, 1981), Manaslu Conservation Area (Devkota et al., 2017), Api Nampa Conservation Area (Khanal et al., 2020), Kangchenjunga Conservation Area (Thapa, 2006 cited in Khanal et al., 2020), and Shey Phoksundo National Park (Thapa, 2006 cited in Khanal et al., 2020). The studies outside of Nepal reported 0.7–7.1 individuals/km^2^ in Bhutan (Wangchuck Centennial National Park, Shrestha et al., 2012 cited in Leki et al., 2018), India (Ladakh region, Fox et al., 1991; Spiti region, Mishra et al., 2004), and China (Qinghai and Gansu Provinces, Schaller et al., 1988; Helan Mountains, Wang et al., 1998).

We consider our minimum density estimates as encouraging for conservation efforts implemented in the Annapurna Conservation Area. In this protected area, wildlife conservation has been pursued through the implementation of an integrated conservation and development program that accounts for local needs in balance with conservation agenda (Baral et al., 2019). Such programs are reported to vary in their effectiveness to protect biodiversity (Newmark & Hough, 2000; Wells et al., 1999), but our study shows that the maintenance of relatively high densities of wildlife, at least blue sheep, appears possible despite ongoing socioeconomic development (Baral et al., 2019). We assume that various favorable conditions including the absence of strong hunting pressure by humans (R.P. Lama & T.R. Ghale, personal communication) and the availability of high‐quality foraging areas (Harris, 2014) are the main causes of high blue sheep density in Manang. Moreover, local people generally have positive attitudes toward blue sheep (Oli et al., 1994). In these conditions, blue sheep may habituate to humans as shown in areas with a large number of pilgrims and tourists (Bhardwaj et al., 2010; Zhang et al., 2013), but may still remain vigilant at the expense of their foraging and resting time budgets (Jiang et al., 2013).

However, this apparent conservation success should not lead to overly optimistic conclusions and complacency, since current developments may threaten the ecosystem and affect blue sheep in the future. There are new projects for infrastructure development in the area, such as construction of roads to remote villages and tourist facilities for ever‐increasing visitor numbers (Baral et al., 2019, R.P. Lama & M. Filla, personal observations). This will further increase the pressure on wildlife populations and habitats, with lag effects to be noticed only after some time (Bürgi et al., 2017). In addition, international tourism as a key component of the integrated conservation and development program implemented in the Annapurna region may not be an ideal long‐term solution as it contributes to climate change through increased carbon emissions (Hall et al., 2013), which threatens mountain wildlife and landscapes (Tse‐ring et al., 2010).

Extrapolation of wildlife densities from total counts does not account for undetected individuals, thus leading to density underestimation (Corlatti et al., 2015; Gaillard et al., 2003). Since previous studies illustrated that detection probability of mountain ungulates may be low even by experienced scientists (Tumursukh et al., 2016; Wingard et al., 2011), our estimates from total counts should be taken as conservative. We expect that the application of double‐observer counts (Nichols et al., 2000), which are commonly applied when monitoring mountain ungulates (e.g., Ghoddousi et al., 2016; Suryawanshi et al., 2012), would produce more accurate density estimates. Distance sampling is a common technique of wildlife counting (Buckland et al., 1993), but its applicability to mountain landscapes is debated (Corlatti et al., 2015; Suryawanshi et al., 2012; Wingard et al., 2011). A preliminary re‐analysis of our study results using a distance sampling approach indicated that the underestimation by minimum densities from total counts could be substantial (M. Filla & R.P. Lama, unpublished data).

Keeping healthy populations of blue sheep is key for the maintenance of ecological balance and conservation of other species in the fragile high‐altitude ecosystem of the Annapurna Conservation Area. The blue sheep is the only medium‐sized herbivore commonly present at high elevations throughout the region and represents the main prey for the snow leopard, thus shaping a high relative density of this threatened predator (Chetri et al., 2017; Gaston & Fuller, 2008; McCarthy et al., 2017; Wegge et al., 2012). The significance of blue sheep as a vital prey resource is likely to increase if local people make more efforts to protect their livestock from depredation. Local absence or low abundance of alternative prey, such as the Himalayan marmot (*Marmota himalayana*) and woolly hare (*Lepus oiostolus*), in Manang adds to an increased dependence of snow leopards on blue sheep abundance (Wegge et al., 2012). Likewise, wolves recently recolonized Manang and they also use this food resource, though not selectively (Chetri et al., 2017; Lama et al., 2017). Therefore, we recommend establishing a regular long‐term monitoring scheme for blue sheep in Manang. Such monitoring programs have been implemented in protected landscapes elsewhere (Zhang et al., 2012), and they can be realized by trained staff to provide reliable information to wildlife managers of the Annapurna Conservation Area. We suggest a monitoring system to be based either on total counts along the systematically placed transects and from vantage points or on double‐observer counts as a standardized method accounting for detection probability (Nichols et al., 2000). Total counts allow for the detection of population changes (Largo et al., 2008; Loison et al., 2006), whereas double‐observer counts yield more reliable abundance and density estimates and enable managers to derive additional conservation parameters, such as the carrying capacity and hotspots for snow leopards (e.g., Aryal et al., 2014b; Khanal et al., 2020; Suryawanshi et al., 2012).

Apart from predation risk, foraging availability and thermal conditions shape the distribution and habitat use of wild ungulates (e.g., van Beest et al., 2012; Hebblewhite & Merrill, 2009), and these parameters seemed to also affect habitat selection by blue sheep in Manang. Our study shows that blue sheep selected habitats mainly on a basis of elevation and land cover indicated by NDVI, both in spring and in autumn. This is in line with other blue sheep studies in Phu Valley in Manang, Nepal (Shrestha & Wegge, 2008b), and in Ladakh, India (Namgail et al., 2009). In our study, blue sheep selected elevations of 4,250–4,550 m a.s.l. in spring and significantly higher elevations in autumn. As elevation is a surrogate of air temperature in the Nepalese Himalaya (Aryal et al., 2016; Mokhov & Akperov, 2006), it affects species distribution by determining snowfall, vegetation phenology, and food availability (Aryal et al., 2014b). Particularly, in Manang blue sheep distribution is limited by forests at lower elevations and by sparsely vegetated barren lands with considerable snow cover at higher elevations (Shrestha & Vetaas, 2009; Shrestha & Wegge, 2008a).

Land cover, which is a proxy for food availability, was the second most important variable determining blue sheep distribution. In our study, the species was closely associated with grasslands and shrublands, which is in line with previous studies (Bhardwaj et al., 2010; Harris, 2014; Shrestha & Wegge, 2008a). This pattern reflects the dietary preference of graminoids and forbs by blue sheep (Aryal et al., 2015b; Liu et al., 2007; Shrestha & Wegge, 2008a). Agricultural land also displayed the NDVI range of habitats selected by blue sheep. However, agricultural fields in the surroundings of settlements were rather infrequently used by blue sheep and did not alter the main model output (Figure S5). Nevertheless, occasional crop‐raiding is possible, mainly of barley (*Hordeum* spp.) and buckwheat (*Fagopyrum* spp.; Baral et al., 2019, R.P. Lama & T.R. Ghale, personal communication), which requires increased attention due to concerns expressed in light of translocation programs (Hanson et al., 2020). Moreover, our study suggests the avoidance of barren lands by blue sheep in both seasons. These lands could be used more frequently during the periods of lower activities, like bedding and resting (Liu et al., 2005a; Liu et al., 2005b; Wilson, 1981) spent in secluded places, but we did not cover these periods during our surveys. Interestingly, occasional spotting of blue sheep in open forests disagrees with the general opinion that, except for the Helan Mountain Range, blue sheep avoid entering forested areas (Harris, 2014).

Contrary to our expectations, we did not find strong evidence of a negative impact of livestock presence/absence on blue sheep distribution in Manang at a fine scale. This is in line with our occasional observations of livestock and blue sheep grazing together (R.P. Lama & M. Filla, personal observations). Theoretically, this result could be affected by non‐detection of livestock if they grazed in secluded places or were released from their night sheds late. But we think that such events were rare and did not influence our main conclusions. Moreover, we assume that livestock densities had a stronger effect on blue sheep than livestock presence/absence. The consideration of livestock densities might have increased model performance, but we could not estimate this parameter from our current data. However, blue sheep–livestock interactions were negative in autumn at larger scales more than 1 km apart (Table S2), thus indicating that livestock can be a serious threat to blue sheep due to habitat loss and fragmentation, disease transmission, and dietary competition (Bhattacharya et al., 2020; Dagleish et al., 2007; Shrestha & Wegge, 2008a; Suryawanshi et al., 2010). Therefore, more knowledge is needed about the relationships between livestock and blue sheep.

## 5 CONCLUSION

This study in the Nepalese Himalaya demonstrated that quite high densities of blue sheep, a key prey species for the threatened snow leopard, have been maintained in an area in which conservation and development agendas have been combined. Moreover, we describe how elevation and land cover shape habitat selection by blue sheep in the absence of strong hunting pressure by humans, which is relevant for blue sheep management, habitat protection, and potential translocation programs. In light of the importance of blue sheep in high‐altitude ecosystems, we suggest to conduct more research on blue sheep–livestock interactions and to establish a standardized blue sheep monitoring program based on total counts and/or double‐observer counts for the benefit of blue sheep and snow leopards.

## CONFLICT OF INTEREST

The authors declare that there is no conflict of interest.

## AUTHOR CONTRIBUTIONS

MF: Conceptualization; formal analysis; funding acquisition; investigation; project administration; visualization; writing—original draft. RPL: Conceptualization; funding acquisition; investigation; project administration; writing—review and editing. TRG: Investigation; writing—review and editing. JS: Formal analysis; visualization; writing—review and editing. TF: Formal analysis; visualization; writing—review and editing. RRA: Formal analysis; writing—review and editing. MH: Conceptualization; funding acquisition; supervision; writing—review and editing. MW: Conceptualization; funding acquisition; supervision; writing—review and editing. NB: Conceptualization; supervision; writing—review and editing. IK: Conceptualization; formal analysis; funding acquisition; supervision; writing—review and editing.

## Supporting information


**Figure S1.** Normalized difference vegetation index (NDVI) of various land‐cover types in Manang based on 203 predefined validation points. The land‐cover types in these locations were distributed throughout the study area and verified by ground‐truthing or analysis of satellite imagery (n_permanent snow_ = 11; n_water body_ = 9; n_barren land_ = 34; n_settlement_ = 19; n_agricultural land_ = 20; n_shrubland_ = 40; n_grassland_ = 43; not shown: n_forest_ = 27).

**Figure S2.** Sensitivity analysis with the inclusion of forested areas. Relative variable importance (%) in generalized additive models (GAMs) describing habitat selection by blue sheep in Manang based on direct observations in spring and autumn.

**Figure S3.** Sensitivity analysis with different sets of random points. Plots of generalized additive models (GAMs) describing habitat selection by blue sheep in Manang based on direct observations in spring. Shown are the smooth terms based on the model presented in the paper (black) in comparison with the models using two different sets of random points (orange, blue).

**Figure S4.** Sensitivity analysis with different sets of random points. Plots of generalized additive models (GAMs) describing habitat selection by blue sheep in Manang based on direct observations in autumn. Shown are the smooth terms based on the model presented in the paper (black) in comparison with the models using two different sets of random points (orange, blue).

**Figure S5.** Sensitivity analysis with the exclusion of locations near settlements (< 500 m). Plots of generalized additive models (GAMs) describing habitat selection by blue sheep in Manang based on direct observations in spring (a) and autumn (b). Shown are the smooth terms for the normalized difference vegetation index (NDVI).

**Figure S6.** Habitat use by blue sheep in Manang. Shown are the frequencies of distances of adult blue sheep to cliff (a), stream (b), settlement (c) and trail (d) based on direct observations in spring and autumn.

TableS1‐S5

## Data Availability

The blue sheep presence locations and the R code used to model blue sheep habitat selection are available at the Dryad Digital Repository https://doi.org/10.5061/dryad.vt4b8gtqc.
