# Supplementary figures and images for: In the shadows of snow leopards and the Himalayas: density and habitat selection of blue sheep in Manang, Nepal
Source: Ecol Evol. 2020 Nov 23;11(1):108–22. doi: 10.1002/ece3.6959 (PMC7790628; doi:10.1002/ece3.6959)

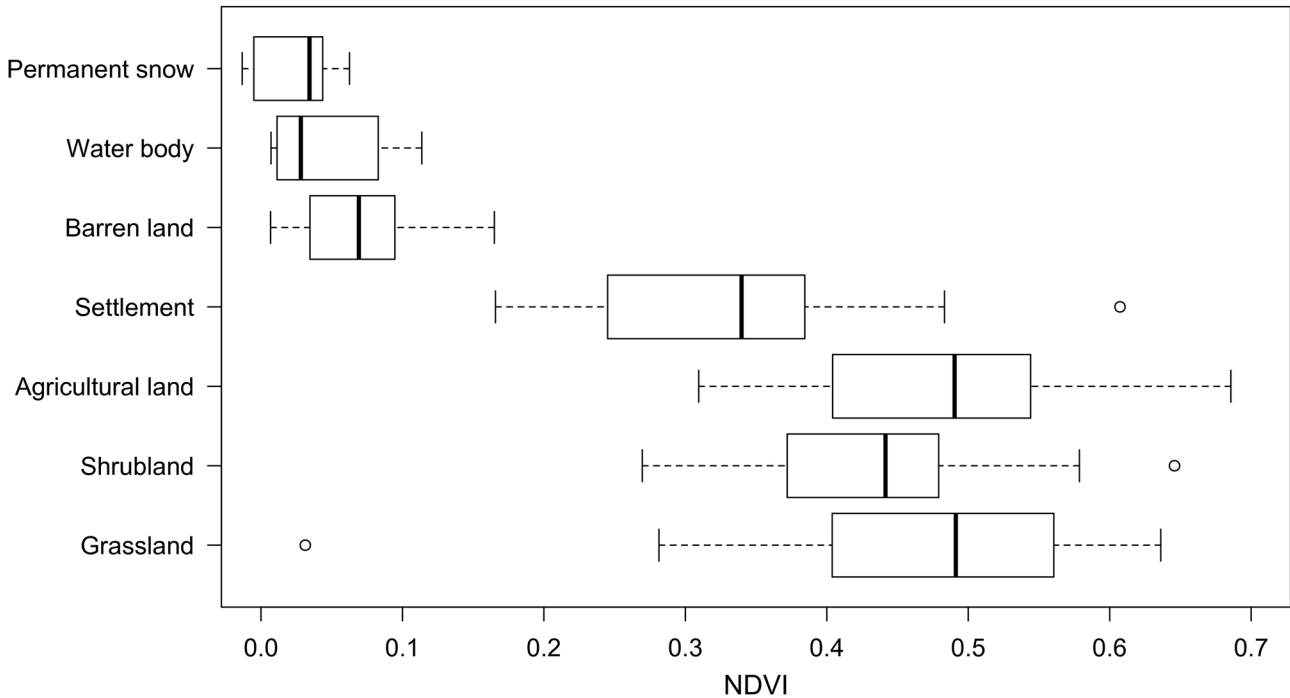

Supplement: Supplementary file 1 — Figure S1. Normalized difference vegetation index (NDVI) of various land‐cover types in Manang based on 203 predefined validation points. The land‐cover types in these locations were distributed throughout the study area and verified by ground‐truthing or analysis of satellite imagery (npermanent snow = 11; nwater body = 9; nbarren land = 34; nsettlement = 19; nagricultural land = 20; nshrubland = 40; ngrassland = 43; not shown: nforest = 27). [file ECE3-11-108-s001.pdf]

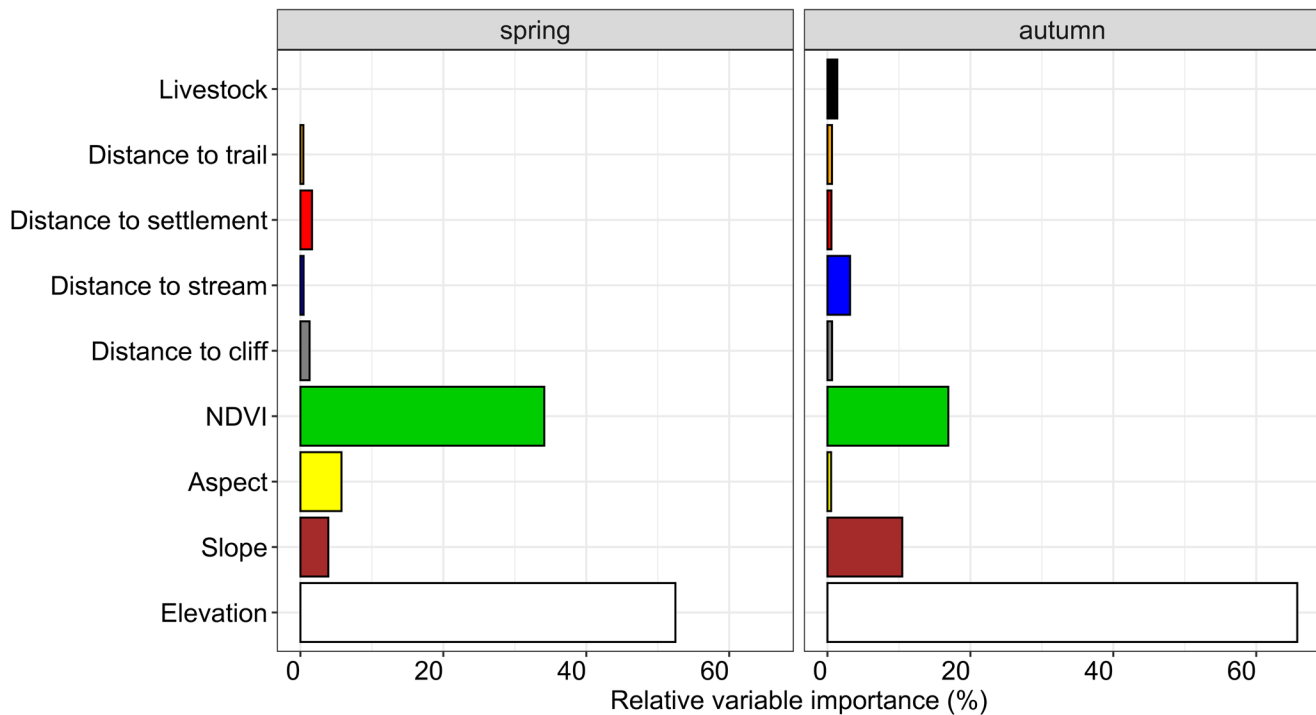

Supplement: Supplementary file 2 — Figure S2. Sensitivity analysis with the inclusion of forested areas. Relative variable importance (%) in generalized additive models (GAMs) describing habitat selection by blue sheep in Manang based on direct observations in spring and autumn. [file ECE3-11-108-s002.pdf]

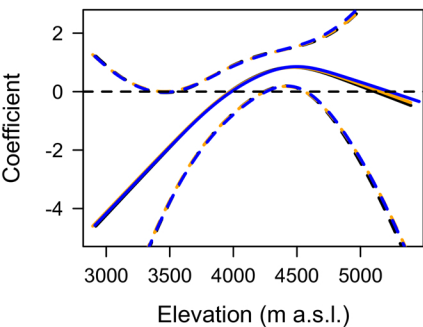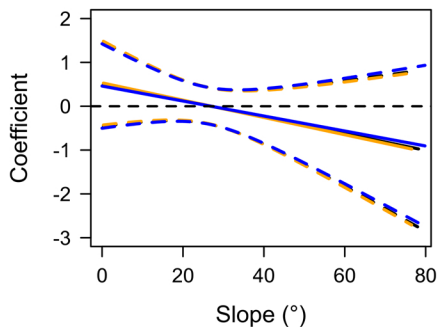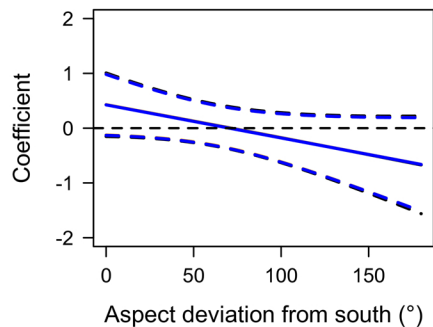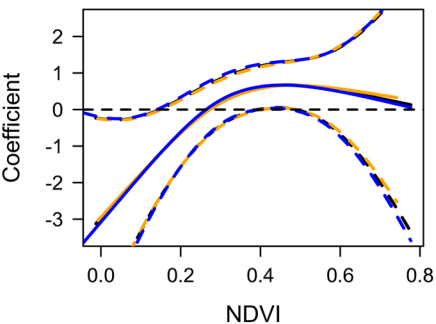

SPRING

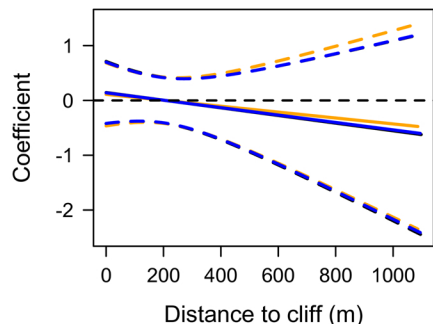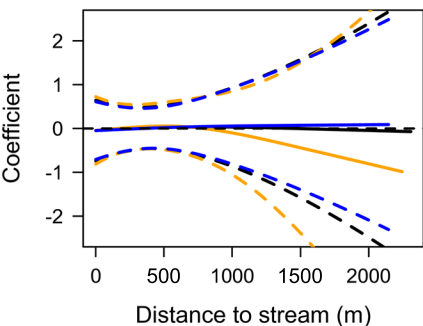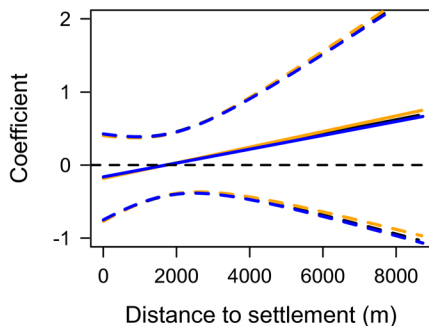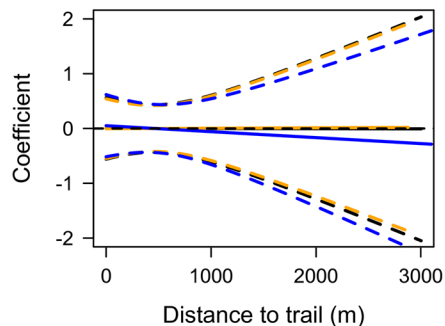

Supplement: Supplementary file 3 — Figure S3. Sensitivity analysis with different sets of random points. Plots of generalized additive models (GAMs) describing habitat selection by blue sheep in Manang based on direct observations in spring. Shown are the smooth terms based on the model presented in the paper (black) in comparison with the models using two different sets of random points (orange, blue). [file ECE3-11-108-s003.pdf]

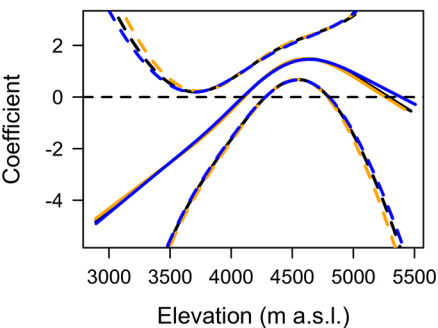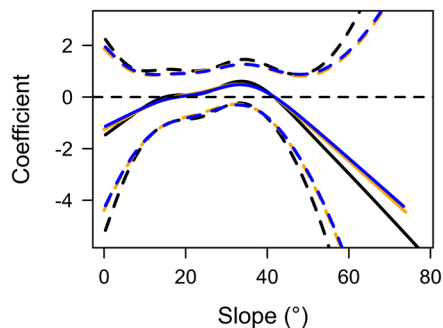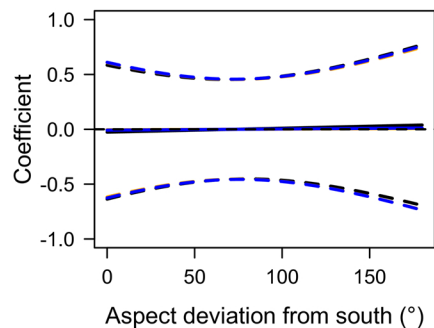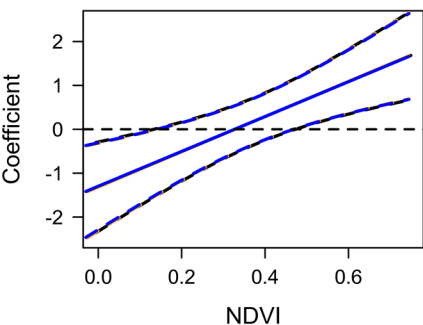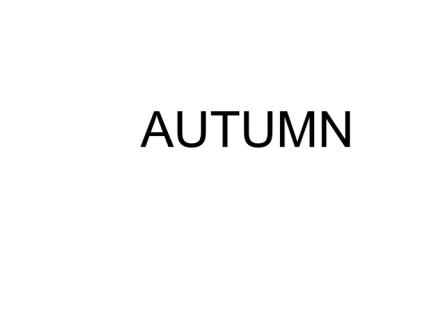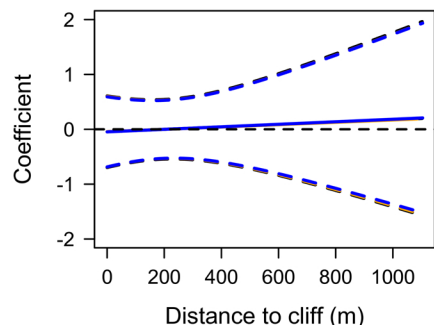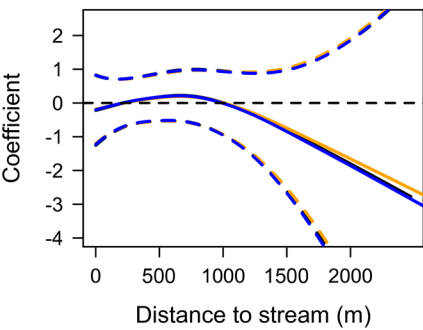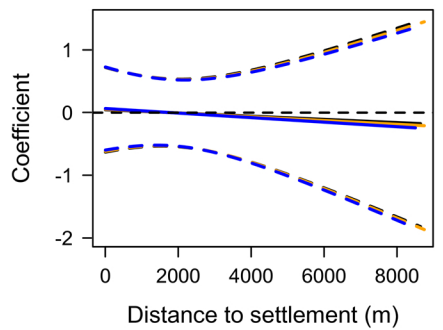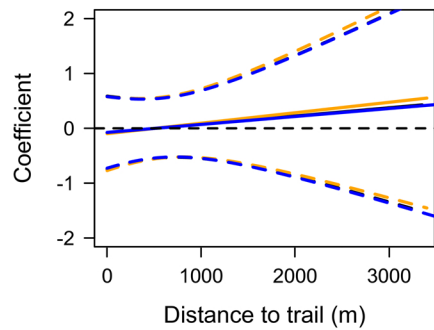

Supplement: Supplementary file 4 — Figure S4. Sensitivity analysis with different sets of random points. Plots of generalized additive models (GAMs) describing habitat selection by blue sheep in Manang based on direct observations in autumn. Shown are the smooth terms based on the model presented in the paper (black) in comparison with the models using two different sets of random points (orange, blue). [file ECE3-11-108-s004.pdf]

(a)

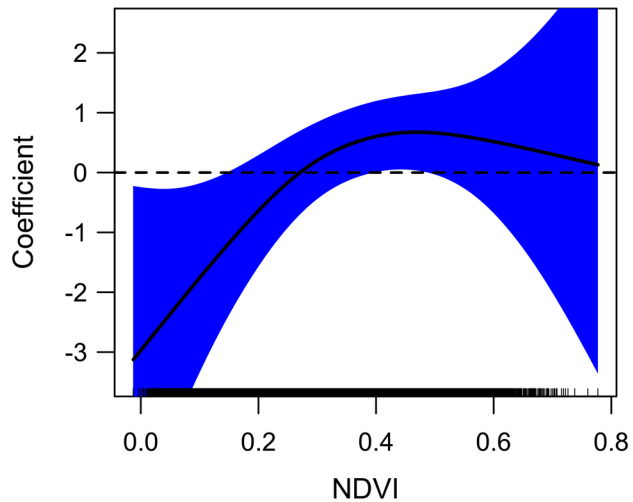

(b)

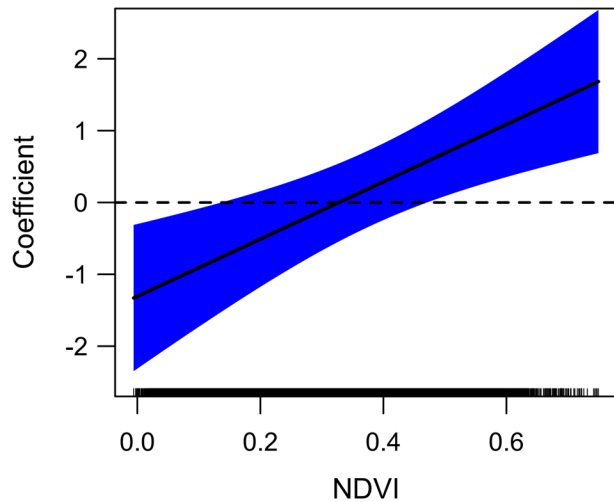

Supplement: Supplementary file 5 — Figure S5. Sensitivity analysis with the exclusion of locations near settlements (< 500 m). Plots of generalized additive models (GAMs) describing habitat selection by blue sheep in Manang based on direct observations in spring (a) and autumn (b). Shown are the smooth terms for the normalized difference vegetation index (NDVI). [file ECE3-11-108-s005.pdf]

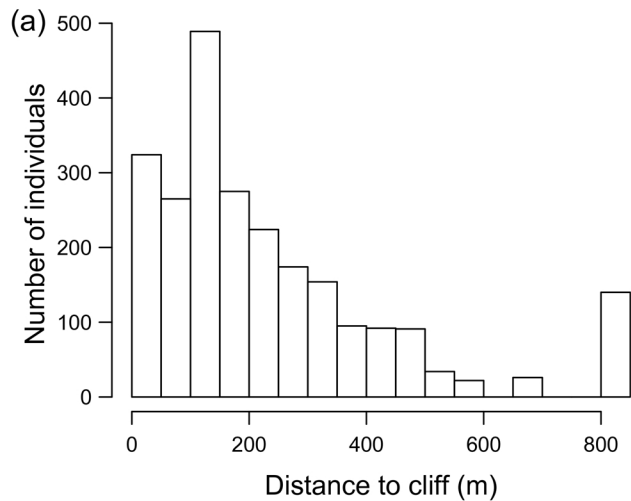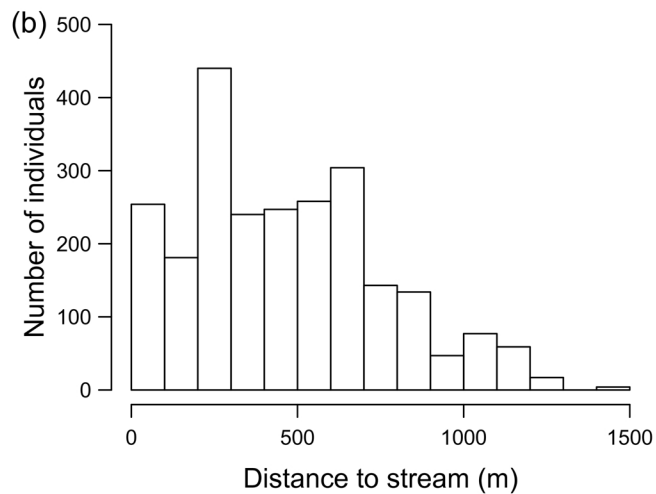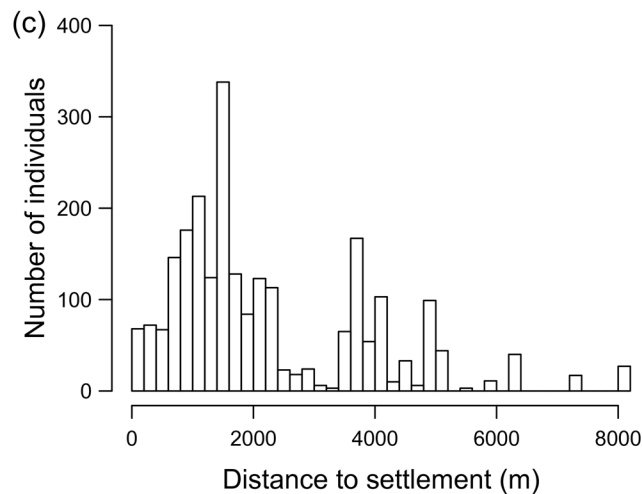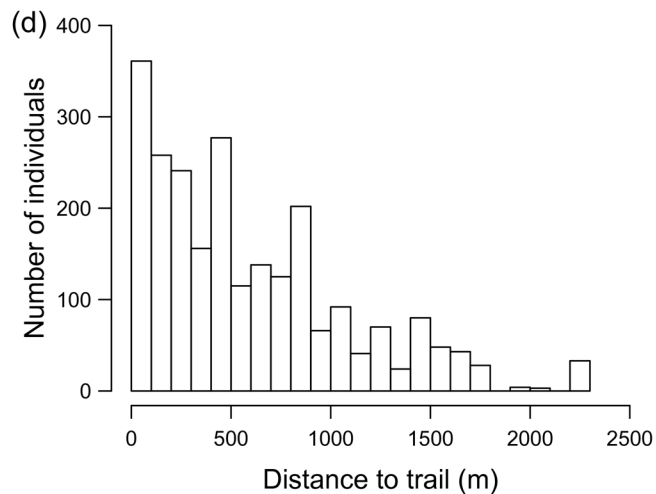

Supplement: Supplementary file 6 — Figure S6. Habitat use by blue sheep in Manang. Shown are the frequencies of distances of adult blue sheep to cliff (a), stream (b), settlement (c) and trail (d) based on direct observations in spring and autumn. [file ECE3-11-108-s006.pdf]
