# Supplementary material for: In the shadows of snow leopards and the Himalayas: density and habitat selection of blue sheep in Manang, Nepal
Source: Ecol Evol. 2020 Nov 23;11(1):108–22. doi: 10.1002/ece3.6959 (PMC7790628; doi:10.1002/ece3.6959)
Supplement: Supplementary file 7 — TableS1‐S5 [file ECE3-11-108-s007.docx]

**Table S1.** P-values of pairwise Wilcoxon rank-sum test of the normalized difference vegetation index (NDVI) of various land-cover types in Manang based on 203 predefined validation points. The land-cover types in these locations were distributed throughout the study area and verified by ground-truthing or analysis of satellite imagery (n_permanent snow_ = 11; n_water body_ = 9; n_barren land_ = 34; n_settlement_ = 19; n_agricultural land_ = 20; n_shrubland_ = 40; n_grassland_ = 43; ^*^not used in the analysis: n_forest_ = 27)

|  | Forest^*^ | Grassland | Shrubland | Agricultural land | Settlement | Barren land | Water body |
| --- | --- | --- | --- | --- | --- | --- | --- |
| Grassland | 0.022 | - | - | - | - | - | - |
| Shrubland | <0.001 | 0.109 | - | - | - | - | - |
| Agricultural land | 0.098 | 1.000 | 0.225 | - | - | - | - |
| Settlement | <0.001 | <0.001 | 0.003 | <0.001 | - | - | - |
| Barren land | <0.001 | <0.001 | <0.001 | <0.001 | <0.001 | - | - |
| Water body | <0.001 | <0.001 | <0.001 | <0.001 | <0.001 | 0.276 | - |
| Permanent snow | <0.001 | <0.001 | <0.001 | <0.001 | <0.001 | 0.017 | 1.000 |

**Table S2**. Sensitivity analysis with alternative buffer widths around livestock locations. Summary of generalized additive models (GAMs) describing habitat selection by blue sheep in Manang based on direct observations in spring and autumn. The estimates of the coefficient, standard error (SE), z-values (z) and p-values (p) are shown for the effects of livestock presence. The asterisk (*) indicates that distance to settlement was excluded from spring models due to high correlation with livestock presence (|r| > 0.7).

| Buffer width (m) | Spring | | | | Autumn | | | |
| --- | --- | --- | --- | --- | --- | --- | --- | --- |
|  | Coefficient | SE | z | p | Coefficient | SE | z | p |
| 100 | 0.232 | 1.211 | 0.192 | 0.848 | 1.009 | 1.376 | 0.733 | 0.464 |
| 250 | 0.770 | 0.515 | 1.495 | 0.135 | -0.252 | 0.702 | -0.359 | 0.720 |
| 500* | 0.165 | 0.413 | 0.400 | 0.689 | -0.444 | 0.436 | -1.019 | 0.308 |
| 750* | 0.558 | 0.401 | 1.392 | 0.164 | -0.588 | 0.393 | -1.497 | 0.135 |
| 1000 | 0.250 | 0.437 | 0.571 | 0.568 | -0.830 | 0.397 | -2.090 | 0.037 |
| 1500* | -0.034 | 0.467 | -0.073 | 0.942 | -1.071 | 0.400 | -2.698 | 0.007 |

**Table S3**. Sensitivity analysis with the inclusion of forested areas. Summary of generalized additive models (GAMs) describing habitat selection by blue sheep in Manang based on direct observations in spring and autumn. The estimates of the coefficient, standard error (SE), z-values (z), and p-values (p) are shown for categorical variables (not given for spring due to multicollinearity). The estimated degrees of freedom (edf), residual degrees of freedom (Ref.df), chi-square test statistics (χ^2^) and p-values (p) are given for continuous variables.

| Variables | Spring | | | | Autumn | | | |
| --- | --- | --- | --- | --- | --- | --- | --- | --- |
| Categorical variables | | | | | | | | |
|  | Coefficient | SE | z | p | Coefficient | SE | z | p |
| (Intercept) | -0.774 | 0.241 | -3.218 | 0.001 | -1.044 | 0.301 | -3.464 | <0.001 |
| Livestock | - | - | - | - | -0.339 | 0.433 | -0.783 | 0.434 |
| Continuous variables | | | | | | | | |
|  | edf | Ref.df | χ^2^ | p | edf | Ref.df | χ^2^ | p |
| Elevation | 2.282 | 2.931 | 13.435 | 0.003 | 2.746 | 3.511 | 24.355 | <0.001 |
| Slope | 1.001 | 1.002 | 1.349 | 0.246 | 2.905 | 3.689 | 5.893 | 0.182 |
| Aspect | 1.071 | 1.137 | 2.878 | 0.119 | 1.000 | 1.001 | 0.006 | 0.939 |
| NDVI | 2.199 | 2.798 | 11.227 | 0.010 | 1.000 | 1.001 | 10.766 | 0.001 |
| Cliff | 1.001 | 1.002 | 0.470 | 0.494 | 1.001 | 1.002 | 0.078 | 0.781 |
| Stream | 1.061 | 1.120 | 0.011 | 0.903 | 1.962 | 2.502 | 2.423 | 0.355 |
| Settlement | 1.000 | 1.001 | 0.540 | 0.463 | 1.000 | 1.000 | 0.025 | 0.875 |
| Trail | 1.001 | 1.002 | 0.000 | 0.999 | 1.001 | 1.001 | 0.108 | 0.743 |

**Table S4**. Estimated blue sheep density in Neshyang Valley and Nar Phu Valley in Manang. Minimum density estimates are extrapolated from total counts and consider various maximum sighting distances for viewshed calculations along the transects.

| Maximum sighting distance (m) | Area | Season | Visible area (km^2^) | Blue sheep counted (N) | Blue sheep density (N/km^2^) |
| --- | --- | --- | --- | --- | --- |
| 500 | Neshyang Valley | spring | 56.0 | 543 | 9.7 |
|  |  | autumn | 59.8 | 384 | 6.4 |
|  | Nar Phu Valley | spring | 43.9 | 704 | 16.0 |
|  |  | autumn | 43.1 | 609 | 14.1 |
| 1000 | Neshyang Valley | spring | 98.6 | 578 | 5.9 |
|  |  | autumn | 102.3 | 679 | 6.6 |
|  | Nar Phu Valley | spring | 81.2 | 809 | 10.0 |
|  |  | autumn | 78.8 | 740 | 9.4 |
| 1500 | Neshyang Valley | spring | 124.3 | 578 | 4.6 |
|  |  | autumn | 127.0 | 766 | 6.0 |
|  | Nar Phu Valley | spring | 108.7 | 830 | 7.6 |
|  |  | autumn | 106.6 | 840 | 7.9 |
| 2000 | Neshyang Valley | spring | 135.6 | 578 | 4.3 |
|  |  | autumn | 139.2 | 766 | 5.5 |
|  | Nar Phu Valley | spring | 116.1 | 830 | 7.1 |
|  |  | autumn | 119.1 | 882 | 7.4 |

**Table S5**. Summary of efforts to monitor blue sheep in Manang based on the maximum sighting distance of 1500 m.

|  |  | **Spring 2019** | | | | **Autumn 2019** | | | |
| --- | --- | --- | --- | --- | --- | --- | --- | --- | --- |
| **Grid cell ID** | **Valley** | **Transect length** (km; [number]) | **View-shed** (km^2^) | **Blue sheep counted** (N) | **Blue sheep density**  (N/km^2^) | **Transect length** (km; [number]) | **View-shed** (km^2^) | **Blue sheep counted** (N) | **Blue sheep density**  (N/km^2^) |
| 595 | Nar Phu | 3.6 [1] | 6.9 | 199 | 29.0 | 4.0 [1] | 7.2 | 152 | 21.0 |
| 619 | Nar Phu | 5.3 [2] | 10.8 | 203 | 18.9 | 6.4 [2] | 11.9 | 194 | 16.3 |
| 620 | Nar Phu | 3.3 [1] | 9.0 | 10 | 1.1 | 4.0 [1] | 9.0 | 185 | 20.6 |
| 635 | Neshyang | 5.5 [1] | 7.6 | 29 | 3.8 | 5.4 [1] | 7.6 | 144 | 19.0 |
| 640 | Nar Phu | 3.2 [1] | 6.8 | 78 | 11.5 | 3.2 [1] | 6.7 | 35 | 5.2 |
| 642 | Nar Phu | 4.4 [2] | 7.5 | 89 | 11.8 | 4.1 [2] | 7.0 | 108 | 15.3 |
| 658 | Neshyang | 2.5 [1] | 6.7 | 80 | 11.9 | - | - | - | - |
| 659 | Neshyang | 7.3 [4] | 9.3 | 170 | 18.3 | 6.2 [3] | 10.7 | 159 | 14.9 |
| 664 | Nar Phu | 4.0 [2] | 10.2 | 111 | 10.9 | 4.7 [2] | 10.4 | 24 | 2.3 |
| 666 | Nar Phu | 3.6 [2] | 8.5 | 37 | 4.4 | 4.1 [2] | 7.1 | 17 | 2.4 |
| 682 | Neshyang | 4.7 [3] | 8.8 | 35 | 4.0 | 5.3 [4] | 9.6 | 58 | 6.0 |
| 683 | Neshyang | 6.3 [3] | 8.4 | 30 | 3.6 | 6.6 [3] | 8.9 | 84 | 9.5 |
| 684 | Neshyang | 4.8 [2] | 9.9 | 71 | 7.2 | 5.2 [2] | 10.8 | 36 | 3.3 |
| 685 | Neshyang | 6.8 [1] | 10.0 | 46 | 4.6 | 8.0 [2] | 11.1 | 61 | 5.5 |
| 686 | Neshyang | 5.0 [3] | 8.4 | 30 | 3.6 | 5.8 [3] | 8.3 | 83 | 10.0 |
| 687 | Neshyang, Nar Phu | 6.7 [4] | 8.2 | 30 | 3.7 | 8.0 [4] | 9.8 | 79 | 8.1 |
| 688 | Nar Phu | 3.5 [2] | 10.4 | 0 | 0.0 | 3.7 [2] | 10.9 | 30 | 2.8 |
| 689 | Nar Phu | 4.9 [5] | 11.4 | 103 | 9.1 | 5.9 [5] | 11.1 | 69 | 6.2 |
| 690 | Nar Phu | 4.2 [2] | 10.0 | 0 | 0.0 | 4.0 [2] | 11.0 | 10 | 0.9 |
| 709 | Neshyang | 2.7 [2] | 2.8 | 0 | 0.0 | 4.4 [3] | 4.9 | 5 | 1.0 |
| 710 | Neshyang | 3.2 [3] | 7.7 | 3 | 0.4 | 4.7 [4] | 9.6 | 20 | 2.1 |
| 711 | Neshyang | 7.9 [2] | 11.1 | 0 | 0.0 | 7.5 [2] | 11.2 | 2 | 0.2 |
| 712 | Neshyang | 4.4 [2] | 7.9 | 28 | 3.5 | 4.6 [2] | 8.3 | 24 | 2.9 |
| 713 | Neshyang | 6.2 [2] | 9.0 | 6 | 0.7 | 5.6 [2] | 7.8 | 11 | 1.4 |
| 715 | Nar Phu | 4.1 [2] | 7.5 | 0 | 0.0 | 4.1 [2] | 7.5 | 16 | 2.1 |
| 736 | Neshyang | 5.0 [3] | 9.1 | 20 | 2.2 | 5.4 [3] | 9.1 | 0 | 0.0 |
| 738 | Nar Phu | 3.8 [1] | 6.3 | 0 | 0.0 | 3.8 [1] | 6.2 | 0 | 0.0 |
| 1001 | Nar Phu | 1.0 [1] | 3.2 | 0 | 0.0 | - | - | - | - |
